# A successful case of complete surgical resection via left upper and right lower lobectomy for bilateral lung metastases of a perivascular epithelioid cell tumor in the colon: a case report

**DOI:** 10.1186/s40792-021-01314-4

**Published:** 2021-10-30

**Authors:** Yoshinobu Fuse, Shohei Mori, Shun Sato, Daiki Kato, Takamasa Shibazaki, Takeo Nakada, Mitsuo Yabe, Hideki Matsudaira, Jun Hirano, Takashi Ohtsuka

**Affiliations:** 1 Division of Thoracic Surgery, Department of Surgery, The Jikei University School of Medicine, 3-25-8 Nishishinbashi, Minatoku, Tokyo, 105-0003 Japan; 2 Department of Pathology, The Jikei University School of Medicine, 3-25-8 Nishishinbashi, Minatoku, Tokyo, 105-0003 Japan

**Keywords:** Perivascular epithelioid cell tumor, Lung metastasis, Bilateral lobectomy

## Abstract

**Background:**

Perivascular epithelioid cell tumors (PEComas) are rare mesenchymal neoplasms with malignant potential. No effective treatment other than surgical resection has been established for lung metastases of PEComas. We describe a patient who underwent complete surgical resection via bilateral lobectomy involving a two-step procedure for lung metastases 8 years after undergoing radical surgery for a colonic PEComa.

**Case presentation:**

A 53-year-old woman underwent partial colectomy for a PEComa in the transverse colon 8 years ago. She presented with an abnormal chest shadow during a health examination. Chest computed tomography (CT) revealed a solid nodule 2 cm in diameter located centrally in the right lower lobe and a solid nodule 3 cm in diameter located centrally in the left upper lobe. Positron emission tomography revealed 18F-fluorodeoxyglucose uptake in these nodules. These nodules were suspected to be metastatic tumors of the colonic PEComa and were considered for complete surgical resection. Segmentectomy could not be performed because of the anatomical location of the tumors straddling the segments; therefore, bilateral lobectomy was required for complete surgical resection. Therefore, we performed two-step lobectomy safely with the expectation of pulmonary function recovery. Microscopically, the tumors were diagnosed as lung metastases of the PEComa. One year after the last surgery, no recurrence was detected, and the patient’s pulmonary function improved.

**Conclusions:**

This case indicates that even if multiple lung metastases of a PEComa require bilateral lobectomy, complete resection with a two-step surgery may be considered.

## Background

Perivascular epithelioid cell tumors (PEComas) are rare mesenchymal neoplasms originating in various organs, including the retroperitoneum, uterus, uterine cervix, gastrointestinal tract, kidneys, liver, breasts, and lungs [1]. Although the natural course of PEComas is not elucidated well, PEComas have malignant potential, and cases of distant metastasis have been reported [2, 3]. The treatment for metastatic recurrences of PEComas after primary lesion resection has not been established; however, radical resection of lung metastases associated with postoperative recurrence after the first radical resection of PEComas has been reported [4–8]. We report a patient who underwent complete surgical resection via left upper and right lower lobectomy involving a two-step procedure for bilateral lung metastases detected 8 years after undergoing radical surgery for a PEComa in the colon.

## Case presentation

**Fig. 1:**
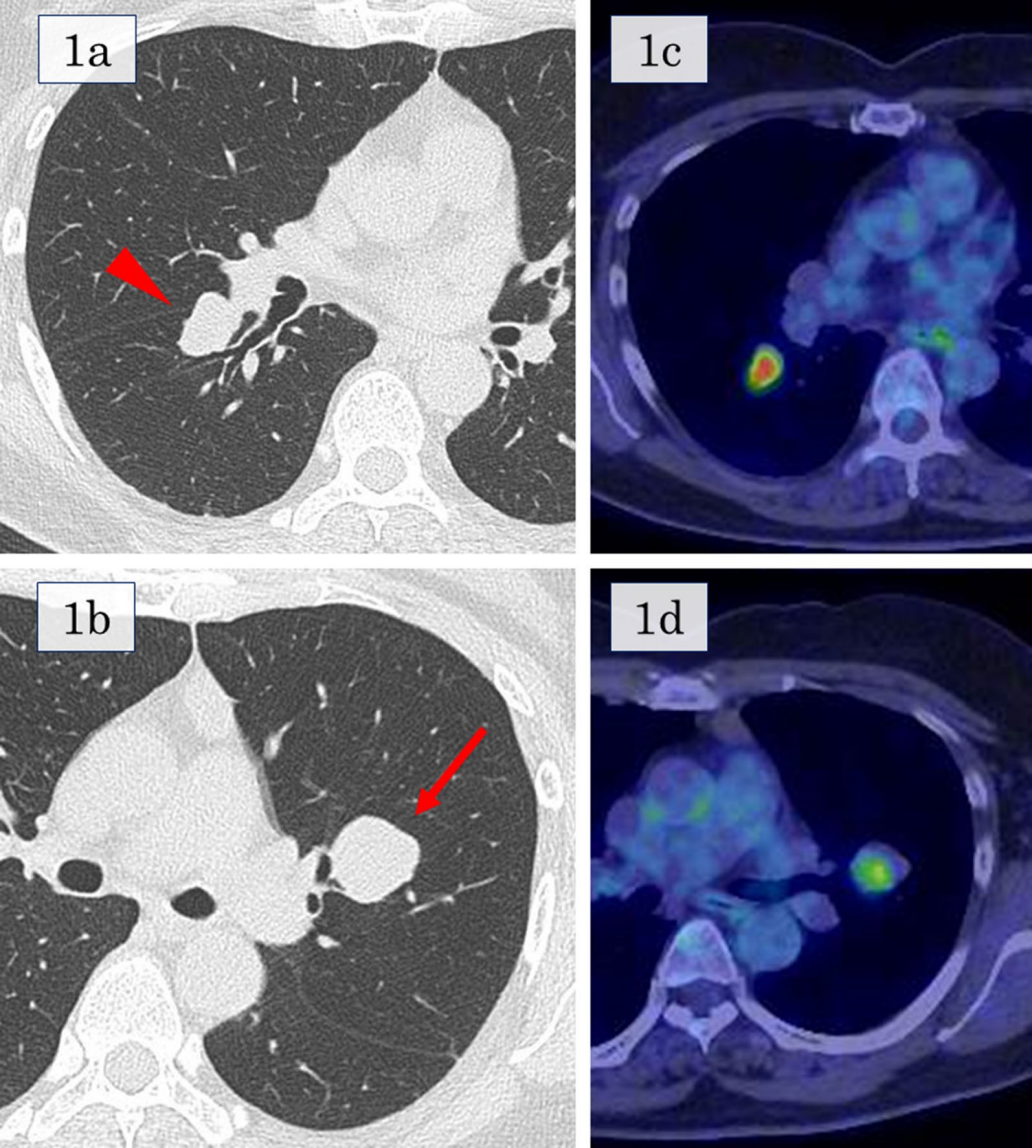
Computed tomography and 18F-fluorodeoxyglucose positron emission tomography findings. **a** A solid nodule (maximum diameter, 2 cm) located centrally in the right lower lobe. **b** A solid nodule (maximum diameter, 3 cm) located centrally in the left upper lobe. **c** Maximum standardized uptake value of 8.3 corresponding to the nodule in the right lower lobe. **d** Maximum standardized uptake value of 5.7 corresponding to the nodule in the left upper lobe

**Fig. 2:**
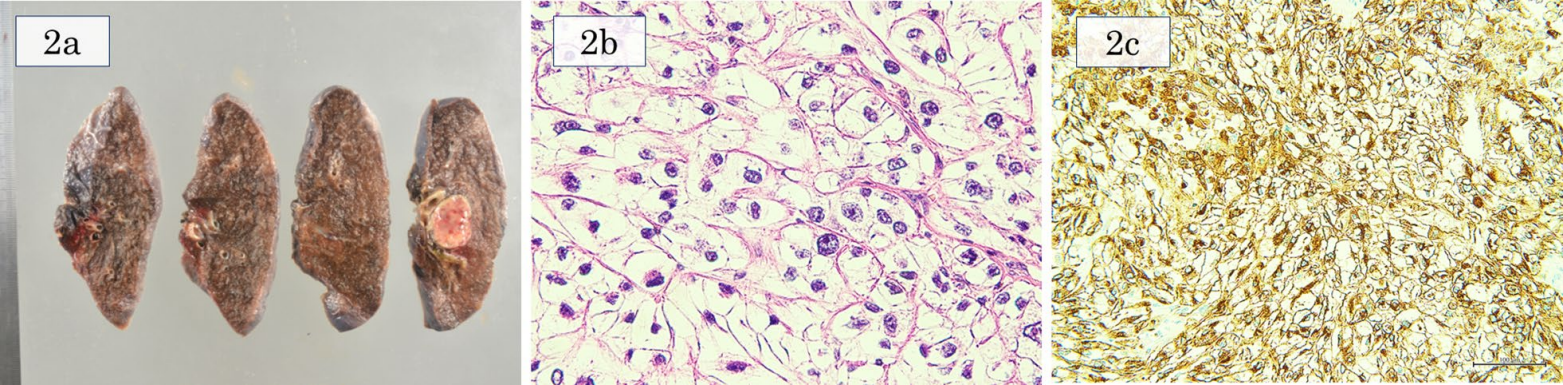
Gross view and microscopic findings of the tumor. **a** Gross view of the tumor shows a white ampullary nodule with well-defined borders. **b** Microscopically, the tumor comprises a solid sheet of epithelioid cells with abundant clear cytoplasm associated with sinusoidal capillaries (hematoxylin and eosin stain, original magnification × 400). **c** Immunohistochemistry reveals that the tumor cells tested positive for melan-A (original magnification × 200)

**Fig. 3:**
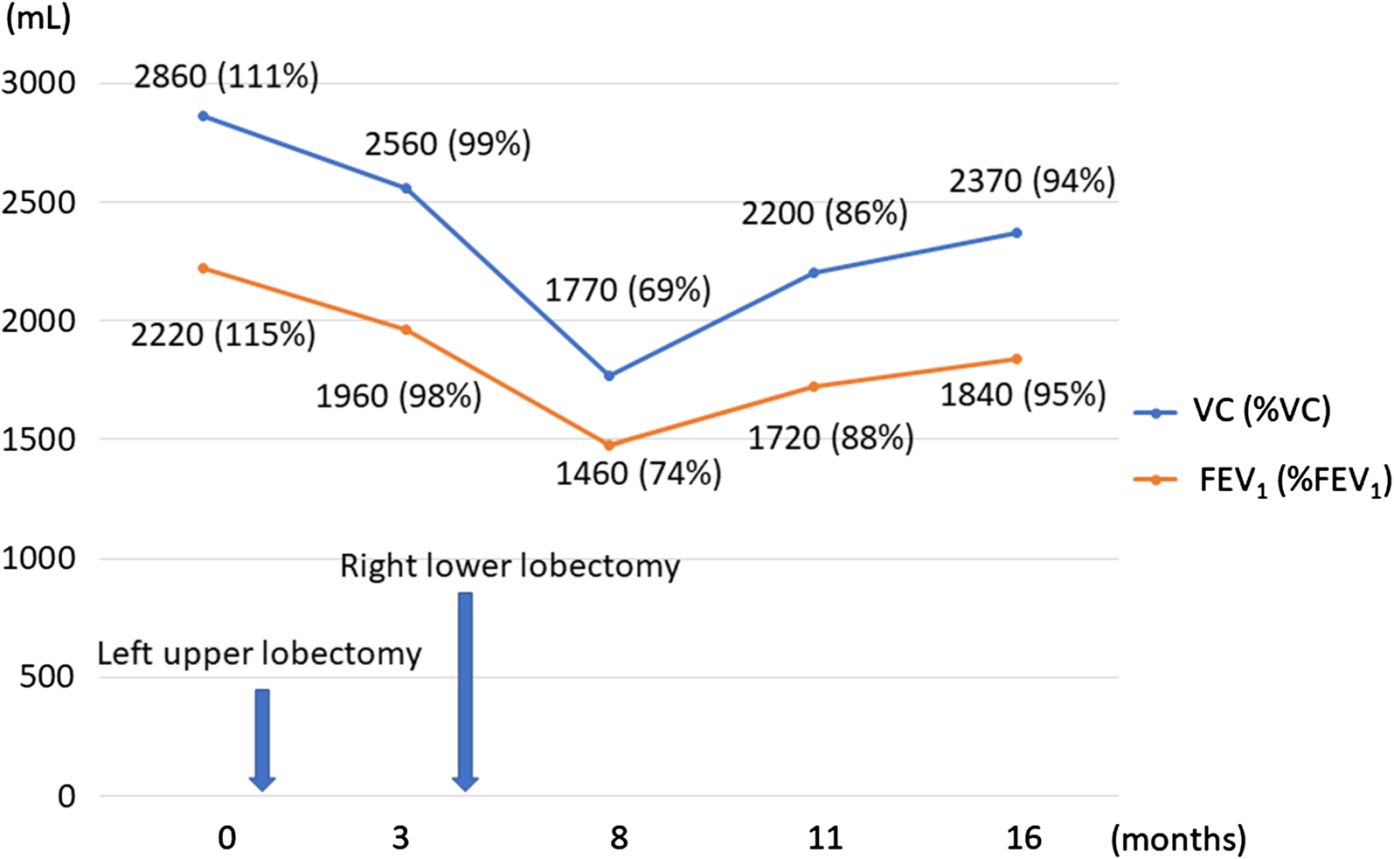
Chronological changes in pulmonary function test results. One year after the second lung surgery, the patient’s pulmonary function is improved, with only a 17% decrease in the % vital capacity and 20% decrease in the % forced expiratory volume in one second compared to the preoperative values

A 53-year-old woman underwent partial colectomy for a submucosal tumor in the transverse colon at another hospital. Histologically, the tumor was diagnosed as a colonic PEComa. Although there was no evidence of recurrence up to 4 years postoperatively, she presented with an abnormal chest shadow during a health examination 8 years postoperatively. She was a never-smoker and had no comorbidities or past medical history. Chest computed tomography (CT) revealed a solid nodule (maximum diameter, 2 cm) located centrally in the right lower lobe and another solid nodule (maximum diameter, 3 cm) located centrally in the left upper lobe (Fig. 1a, b). 18F-fluorodeoxyglucose-positron emission tomography revealed maximum standardized uptake values of 8.3 and 5.7 in each lesion (Fig. 1c, d). These nodules were suspected to be metastatic tumors of the colonic PEComa and were considered for complete surgical resection. Segmentectomy could not be performed because of the anatomical location of the tumors straddling the segments; therefore, bilateral lobectomy was required for complete surgical resection. We considered that the patient would tolerate bilateral lobectomy because the predicted postoperative % forced expiratory volume in one second (FEV_1_) was 56%. For safety, we planned to perform a two-step surgery. First, we performed thoracoscopic left upper lobectomy for the larger lesion located in the left upper lobe. The ports were made on the anterior axillary line in the 3rd and 5th intercostal space and the posterior axillary line in the 4th and 7th intercostal space. The gross view of the surgically resected specimen revealed a white ampullary nodule with well-defined borders (Fig. 2a). Microscopically, the tumor comprised a solid sheet of epithelioid cells with abundant clear cytoplasm associated with sinusoidal capillaries (Fig. 2b). Immunohistochemistry assessment revealed that the tumor cells tested positive for melan-A (Fig. 2c), human melanoma black-45 (HMB-45), caldesmon, and S100 and negative for cluster of differentiation (CD)56, chromogranin A, synaptophysin, AE1/AE3, desmin, α-smooth muscle actin, CD34, CD117, and CAM5.2. Based on these findings, the tumor was diagnosed as lung metastasis of the PEComa. The consistent immunohistochemical staining results with the primary tumor of the colon were positive for HMB-45, caldesmon, and S100 and negative for desmin and α-smooth muscle actin. Expecting an improvement in pulmonary function, we performed right lower lobectomy 3 months later. The predicted postoperative %FEV_1_ was 63% at the time of the second lung surgery. Re-evaluation using CT immediately before the second lung surgery revealed that the right tumor had grown from 2 cm to 2.8 cm in diameter. Although bilateral lung ventilation was required several times because of hypoxia during surgery, the patient underwent right lower lobectomy through a lateral thoracotomy without complications. The tumor in the right lower lobe was also pathologically diagnosed as lung metastasis of the PEComa. One year after the surgery, no recurrence was detected, and the patient’s pulmonary function improved, with only a 17% decrease in vital capacity and 20% decrease in the FEV_1_ compared to the preoperative values. Figure 3 shows the chronological changes in the pulmonary function test results.

## Discussion

We identified two important clinical issues in the present case. Lung metastasis after radical resection of a primary lesion of a PEComa should be treated with complete surgical resection, if possible. In cases of multiple lung metastases requiring bilateral lobectomy, we recommend a safe two-step bilateral lobectomy.

First, lung metastasis after radical resection of a primary lesion of a PEComa should be treated with complete surgical resection, if possible. PEComa has malignant potential, and recurrence and distant metastases have been reported [2, 3]. Common metastatic sites are the liver, lymph nodes, lungs, and bone [2]. Although chemotherapy and mammalian target of rapamycin inhibitors have also been applied in a few cases, a standardized treatment strategy for lung metastasis of PEComas is not yet established [9]. At this time, surgical resection is considered the mainstay of treatment for resectable lung metastasis of PEComas [4–7]. Therefore, complete surgical resection for lung metastasis of PEComas is the appropriate treatment if conditions such as the general patient status and anatomical location of the metastasis permit.

Second, in cases of multiple lung metastases requiring bilateral lobectomy, we recommend a safe two-step approach. In general, if the predicted postoperative %FEV_1_ and % diffusing capacity for carbon monoxide values are both > 60%, the patient is considered at low risk of anatomic lung resection [10]. We considered bilateral lobectomy with a single step to confer rise of postoperative complication risk because the predicted postoperative %FEV_1_ was 56%. Thus, a two-step resection strategy was selected. First, left upper lobectomy of the larger lesion was performed. Three months later, we confirmed that the actual pulmonary function was better, the predicted postoperative %FEV_1_ was 63%, than the initial value and safely performed contralateral lobectomy. The postoperative pulmonary function was superior to the predicted values at any point in time. Takahashi et al. reported that never-smokers showed significantly greater compensatory response than smokers after major lung resection [11]. We believed that the factor of never-smoking related to the superior postoperative pulmonary function to the predicted values at both of times after first and second lung resections. We suggest that two-step resection strategy has the advantage of allowing gradual assessment of surgical tolerance. On the other hand, Toufektzian et al. reported that a pneumonectomy followed by contralateral lobectomy had high mortality rate, 33% [12]. We consider that a pneumonectomy combined with contralateral lobectomy should be avoided.

In this case, the histological feature and immunohistochemical staining results of the primary tumor of the colon and bilateral lung metastases were all consistent with the typical findings of PEComas. PEComas histologically comprise rounded or oval cells with abundant clear or eosinophilic cytoplasm and thin-walled sinusoidal vessels are characteristics [1]. PEComas stain most consistently for HMB-45, melan-A, and microphthalmia transcription factor and may also stain for S100 [1]. All tumors of this case were positive for HMB-45, caldesmon, and S100 and negative for desmin and α-smooth muscle actin. Although bilateral lung metastases were positive for melan-A, primary tumor of the colon had not been performed immunohistochemical staining for melan-A.

In addition, the time from initial radical resection of primary lesions of PEComas to recurrence or metastasis varies, and there remains no consensus on the duration of follow-up. Late recurrences have been reported up to 5 or 7 years after surgery [2, 4]. In the present case, the follow-up was completed up to 4 years after the initial surgery, and lung metastases were detected via a health examination 8 years postoperatively. Although the appropriate follow-up duration is a matter for future debate, we believe that a longer follow-up is desirable.

## Conclusions

PEComas are rare tumors, and no effective treatment other than surgical resection has been established for lung metastases. Complete resection with a two-step surgery may be considered even if multiple lung metastases require bilateral lobectomy.

## Data Availability

Not applicable.

## Abbreviations

PEComa: Perivascular epithelioid cell tumor
CT: Computed tomography
FEV_1_: Forced expiratory volume in one second
HMB-45: Human melanoma black-45
CD: Cluster of differentiation
